# Prediction of preeclampsia and induced delivery at <34 weeks gestation by sFLT-1 and PlGF in patients with abnormal midtrimester uterine Doppler velocimetry: a prospective cohort analysis

**DOI:** 10.1186/1471-2393-14-292

**Published:** 2014-08-28

**Authors:** Johannes Stubert, Stefanie Ullmann, Michael Bolz, Thomas Külz, Max Dieterich, Dagmar-Ulrike Richter, Toralf Reimer

**Affiliations:** Department of Obstetrics and Gynecology, University of Rostock, Suedring 81, 18059 Rostock, Germany; Centre of Prenatal Diagnosis “Praxiszentrum Frauenheilkunde”, Suedring 81, 18059 Rostock, Germany

**Keywords:** Angiogenic factors, PlGF, sFLT-1, Preeclampsia, Sensitivity, Specificity

## Abstract

**Background:**

Women with bilateral abnormal uterine artery Doppler velocimetry (UtADV) are at increased risk for an adverse pregnancy outcome. This study aimed to determine if additional assessment of midtrimester angiogenic factors improves the predictive accuracy of Doppler results for various outcome parameters.

**Methods:**

Women with a bilateral abnormal UtADV, which was defined as a postsystolic incision and/or an increased pulsatility index greater than the 95^th^ centile, and a singleton pregnancy were prospectively recruited between 19 + 0 and 26 + 6 weeks of gestation. Maternal serum levels of placental growth factor (PlGF) and soluble fms-like tyrosine kinase-1 (sFLT-1) were measured with a fully automated immunoassay and their ratio was calculated.

**Results:**

Angiogenic factors could predict the development of preeclampsia (PE), as well as induced delivery at <34 weeks of gestation, but failed to predict the development of normotensive intrauterine growth restriction. Twelve (24.0%) of the 50 recruited women developed PE. Nine of these patients had early-onset disease (<34 + 0 weeks). Six (12.0%) patients were delivered at <34 + 0 weeks. The most useful test results in the prediction of PE and induced delivery at <34 + 0 weeks were observed using the sFLT-1/PlGF >95^th^ centile ratio with a sensitivity, specificity, positive predictive value, and negative predictive value (NPV) of 66.7%, 89.5%, 66.7%, and 89.5% for PE, and 85.7%, 86.1%, 50.1%, and 97.4% for induced delivery, respectively. Positive and negative likelihood ratios were 6.33 (95% CI 2.31–17.38) and 0.37 (95% CI 0.17–0.84) for PE, and 6.14 (95% CI 2.76–13.69) and 0.17 (0.03–1.02) for induced delivery, respectively. Corresponding odds ratios were 17.0 (95% CI 3.5–83.0) and 37.0 (95% CI 3.8–363.9), respectively.

**Conclusions:**

Measurement of angiogenic factors improves the specificity of an abnormal UtADV for prediction of PE. Compared with prediction of PE an abnormal sFLT-1/PlGF ratio revealed higher sensitivity for prediction of induced delivery at <34 + 0 weeks. The NPV of 97% will help to reassure most patients with an abnormal UtADV and a normal sFLT-1/PlGF ratio.

**Electronic supplementary material:**

The online version of this article (doi:10.1186/1471-2393-14-292) contains supplementary material, which is available to authorized users.

## Background

The estimated incidence of preeclampsia (PE) is between 0.4–2.8% of all pregnancies in Europe [1, 2]. Only 15–20% of PE is present a severe clinical course [3]. The majority of severe PE develops early in pregnancy (<34 weeks of gestation) and is frequently associated with a serious maternal and foetal outcome [4–6].

Angiogenic factors have a large effect on development of preeclamptic syndrome [7]. The extent of the antiangiogenic shift, characterized by an increase in antiangiogenic soluble fms-like tyrosine kinase (sFlt)-1 and a decrease in angiogenic placental growth factor (PlGF), correlates with disease severity and precedes the clinical manifestation for several weeks [8–11]. Therefore, analysis of maternal sFLT-1 and PlGF can improve the diagnostic accuracy for detection of PE and allows an estimation of the clinical disease severity [12]. Moreover, removal of sFLT-1 from the maternal circulation by apheresis improves the severity of the disease and consequently prolongs the duration of pregnancy [13].

Although definitive treatment of PE is possible by delivery of the placenta alone, early prediction of high-risk patients may enhance the patient´s care before disease manifestation and could help to reduce mortality and morbidity of the mother and her foetus. A widely used noninvasive approach for identifying high-risk patients comprises the performance of midtrimester Doppler ultrasound measurements of the uterine arteries. An abnormal uterine artery Doppler velocimetry (UtADV) during the second trimester has shown detection rates for PE between 40% and 80% in patients at low risk [14]. Generally, sensitivity is better for the detection of severe and/or early-onset PE compared to mild and late onset cases, but positive predictive accuracy is quite low, and the majority of patients with an abnormal UtADV will not develop PE [15]. However, additional analysis of angiogenic markers may improve the test accuracy for detecting PE. Our prospective study aimed to evaluate sFLT-1 and PlGF as predictive markers for PE in a high-risk collective as identified by an abnormal UtADV in the second trimester.

## Methods

### Selection and inclusion criteria of patients

Patients with a bilateral abnormal UtADV between 19 + 0 and 26 + 6 weeks of gestation were included in this prospective cohort study. The clinical trial was conducted at the Department for Obstetrics and Gynaecology of the University of Rostock, Germany in cooperation with the outpatient centre of prenatal diagnosis “Praxiszentrum Frauenheilkunde” in Rostock between February 2011 and July 2013. The study was approved by the institutional review board of the University of Rostock (IRB No. A2010100) and written informed consent was obtained from all participating patients. An abnormal UtADV was assumed if a bilaterally increased pulsatility index (PI) greater than the 95^th^ centile and/or a distinct postsystolic incision (“notch”) were detected [16]. All measurements were performed by two experienced observers following the recommendations for Doppler ultrasonography measurements in obstetrics [17]. For ultrasound examinations, the Voluson 730, Voluson G8 (both GE Medical Systems, Milwaukee, WI, USA), and HDI5000 SonoCT (Philips Medical Systems, Bothell, WA, USA) were used. Gestational age was calculated from the first day of the last menstrual period and was corrected by ultrasound if measurements of the crown-rump-length during the first trimester revealed a difference of more than 7 days. PE was defined as being present when blood pressure was ≥140/90 mmHg (taken twice, 6 h apart) combined with proteinuria ≥300 mg in a 24-h collection [18]. Inclusion of patients with chronic hypertension (blood pressure level ≥140/90 mmHg prior to 20 weeks of gestation) was possible. In these cases, new onset of proteinuria ≥300 mg per day was regarded as superimposed PE. According to the clinical manifestation, PE was assigned to early onset (<34 weeks of gestation) or late onset (≥34 weeks) [19]. Haemolysis, elevated liver enzymes, and low platelets (HELLP) syndrome was defined as being present, when the platelet count was less than 100 × 10^9^/L (normal range: 150–450 × 10^9^/L), haptoglobin serum levels were below 0.3 g/L (normal range: 0.3–2.0 g/L), and aspartate transaminase (AST) levels were >70 U/L (normal range: 3–34 U/L). Only singleton pregnancies were included. Exclusion criteria were maternal age <18 years, on-going therapy with glucocorticoids or non-steroidal analgesics (with exception of aspirin 100 mg daily), and the presence of anti-phospholipid syndrome.

Small for gestational age (SGA) was defined as a birth weight less than the 10^th^ centile for gestational age according to Voigt et al. [20]. Intrauterine growth restriction (IUGR) was assumed when a SGA birth weight was combined with at least one of the following criteria: (1) oligohydramnios with an amniotic fluid index <5 cm, (2) persistent bilateral abnormal UtADV, (3) a pathological cardiogram, (4) a decrease in foetal growth by ultrasound biometry (“crossing centiles”), and (5) increased PI of the umbilical artery greater than the 95^th^ centile. Because clinicians were not aware of biomarker results, an effect of decision making for the obstetric approach was excluded.

### Sample collection and immunoassays

From each study patient, 2 × 7.5 mL of venous blood was taken at the time of Doppler measurement using a serum collection tube (Sarstedt, Nümbrecht, Germany). The blood clot was immediately separated by centrifugation at 2000 × g for 10 min at 15°C and serum was stored in 1-mL aliquots at -80°C until further investigations were performed. Serum concentrations of PlGF and sFLT-1 were measured with the fully automated Elecsys® sandwich immunoassays (Roche Diagnostics, Penzberg, Germany) based on streptavidin-biotin technology. The assays were performed using the Roche immunoanalyser Elecsys® 2010/cobas e 411 according to the recommendations of the manufacturer. Within and between run coefficients of variation were below 4% for sFLT-1 > 60 pg/mL and PlGF >20 pg/mL. Functional sensitivity was <5 pg/mL [21, 22]. Results were classified using gestational age-dependent centile curves that were provided by the manufacturer and based on data from Verlohren and colleagues [10]. Results were regarded as pathological if sFLT-1 or the ratio of sFLT-1/PlGF was greater than the 95^th^ centile and/or PlGF was less than the 5^th^ centile.

### Statistical analysis

All data were stored and analysed using the IBM SPSS statistical package 19.0 (SPSS Inc. Chicago, IL, USA) and Excel 2010 (Microsoft Corporation, Redmond, WA, USA). Descriptive statistics were computed for continuous and categorical variables. The statistics included mean, median, standard deviation (SD), minimum, maximum, and number of available observations of continuous variables. Values of continuous variables are presented as mean ± SD. For categorical variables, frequencies and relative frequencies are shown. Testing for differences of continuous variables between the groups was accomplished by the ANOVA or the Kruskal–Wallis test, as appropriate. Test selection was based on evaluation of the variables for normal distribution by using the Kolmogorov–Smirnov test. Comparison between the groups for categorical variables was performed using the chi-square test. All P values resulted from two-sided statistical tests and values of P < 0.05 were considered as statistically significant. Criteria of diagnostic validity for sFLT-1 and PlGF alone, as well as the sFLT-1/PlGF ratio, were computed and presented as sensitivity, specificity, positive predictive value (PPV), negative predictive value (NPV), positive likelihood ratio, and negative likelihood ratio. Furthermore, receiver operating characteristic (ROC) curves were calculated and the areas under the curves (AUCs) were reported. The most useful cut-off values were calculated by identification of the highest score for sensitivity and specificity. For the sFLT-1/PlGF ratio >95^th^ centile, odds ratios (OR) were computed for various outcome parameters. The logistic regression model was used to assess the independence of specific outcome parameters. Variables yielding P values ≤0.05 in univariate analysis were entered in the multivariate model and adjusted for maternal body mass index, parity, onset of PE, existence of chronic hypertension, week of gestation at the moment of blood sampling, and use of acetylsalicylic acid. We performed post hoc analysis for estimation of the statistical power of our study collective by logistic regression analysis using G*Power 3.1.9.2 [23]. One-tailed power analysis for prediction of PE by the sFLT-1/PlGF ratio with an α error of 0.05 revealed a statistical power of 95.5% for our sample size.

## Results

### Patients’ characteristics

The patients’ characteristics are shown in Table 1 with the following prevalence of subgroups: PE was found in 24.0% (12/50) of patients, IUGR in 12.0% (6/50), gestational hypertension in 32.0% (16/50), and others in 32.0% (16/50). Subgroup analysis did not show any differences between maternal characteristics, such as age, gravidity, parity, and body mass index. In contrast, significant intergroup differences were found regarding foetal/neonatal parameters. More than 40% of all preeclamptic newborns showed a SGA birth weight. Newborns in the PE subgroup also presented with the lowest mean gestational age. Consequently, the mean birth weight in cases of PE was significantly lower compared with that in the other subgroups, including newborns with normotensive IUGR. Delivery at <34 + 0 weeks occurred in 14.0% (7/50) of all patients. Five of them (71.4%) had PE and two had severe hypertension without proteinuria. All of these patients had an induced delivery because of a severe clinical course indicated by the physicians on duty. One patient with chronic hypertension and concomitant diabetes mellitus type II had an intrauterine foetal death at 26 weeks of gestation. Induction of lung maturation with betamethasone was not performed in three newborns with induced delivery at <34 + 0 weeks, including the patient with intrauterine foetal death. Doppler measurements were repeated in all of the patients after 4 weeks. An abnormal UtADV normalized in 32.0% (16/50) of all cases. There were significant differences between subgroups (P = 0.047) with normalization of an abnormal uterine Doppler in 0.0% of PE (0/12), 33.3% of IUGR (2/6), 31.3% of gestational hypertension (5/16), and 56.3% in the remaining patients (9/16).

**Table 1:** **Maternal and foetal characteristics of the study population**

Characteristic	All patients (n = 50)	PE (n = 12)	IUGR (n = 6) ^#^	GH (n = 16)	Others (n = 16)	P-value
**Age, y**						
Mean ± SD	29.7 ± 4.9	29.8 ± 3.9	33.3 ± 4.8	28.3 ± 3.6	29.6 ± 6.3	0.206*
Minimum - maximum	19 - 40	24 - 38	28 - 40	22 - 34	19 - 39	
**Gravidity**						0.613**
Median	1	1	2	1	2	
Minimum - maximum	1 - 9	1 - 9	1 - 8	1 - 5	1 - 5	
**Parity**						0.612**
Median	0	0	0	0	0	
Minimum - maximum	0 - 5	0 - 4	0 - 5	0 - 2	0 - 1	
**Body mass index (pregravid), kg/m** ^**2**^						0.132*
Mean ± SD	27.1 ± 8.2	30.6 ± 8.0	21.7 ± 3.2	28.1 ± 9.1	25.6 ± 6.3	
Minimum - maximum	14.5 - 47.0	19.8 - 45.0	18.7 - 27.3	14.5 - 45.7	18.8 - 47.0	
**Chronic hypertension, n (%)**	8 (16.0%)	4 (33.3%)	0 (0.0%)	4 (25.0%)	0 (0.0%)	0.049***
**Thrombophilia, n (%)**	5 (10.0%)	0 (0.0%)	0 (0.0%)	3 (18.8%)	2 (12.5%)	0.755***
**Nicotine abuse, n (%)**	14 (28.0%)	5 (41.7%)	1 (16.7%)	6 (37.5%)	2 (12.5%)	0.238***
**Highest systolic blood pressure, mmHg**						< 0.001*
Mean ± SD	143.2 ± 23.1	165.8 ± 21.2	128.7 ± 5.4	149.4 ± 20.0	124.7 ± 9.9	
Minimum - maximum	110 - 200	140 - 200	120 - 135	130 - 200	110 - 140	
**Highest diastolic blood pressure, mmHg**						< 0.001*
Mean ± SD	90.4 ± 17.6	110.5 ± 13.4	79.7 ± 6.2	91.8 ± 15.9	77.3 ± 7.1	
Minimum - maximum	60 - 130	90 - 130	70 - 86	60 - 124	60 - 90	
**Proteinuria, mg/d**						
Mean ± SD	1180 ± 2524	2613 ± 3394	n.a.	157 ± 196	n.a.	
Minimum - maximum	0 - 9986	109 - 9986	n.a.	0 - 283	n.a.	
**Placenta weight, g**						0.129*
Mean ± SD	324 ± 98	300 ± 86	322 ± 29	286 ± 145	443 ± 71	
Minimum - maximum	124 - 510	182 - 461	283 - 364	124 - 476	369 - 510	
**Amnion fluid index (AFI), n (%)**						0.839***
Oligohydramnion (AFI ≤ 8)	13 (26.0%)	3 (25.0%)	2 (33.3%)	4 (25.0%)	4 (25.0%)	
Polyhydramnion (AFI > 18)	7 (14.0%)	3 (25.0%)	1 (16.7%)	1 (6.3%)	2 (12.5%)	
**Birth weight, g**						< 0.001*
Mean ± SD	2588 ± 809	1859 ± 658	2270 ± 303	2588 ± 750	3175 ± 626	
Minimum - maximum	620 - 3940	890 - 2980	1750 - 2620	620 - 3610	1860 - 3940	
**Percentile of birth weight, n (%)**						
SGA (<10^th^ centile)	16 (32.0%)	5 (41.7%)	6 (100.0%)	6 (37.5%)	0 (0.0%)	
LGA (>90^th^ centile)	0 (0.0%)	0 (0.0%)	0 (0.0%)	0 (0.0%)	0 (0.0%)	
**5´-APGAR-Score**						0.398**
Median	10	9	10	10	10	
Minimum - maximum	8 - 10	8 - 10	9 - 10	8 - 10	9 - 10	
**Induction of lung maturation, n (%)**	13 (26.0%)	4 (33.3%)	6 (100.0%)	2 (12.5%)	1 (6.3%)	0.019***
**Newborns < 34 + 0 weeks, n (%)**	7 (14.0%)	5 (41.7%)	0 (0.0%)	2 (12.5%)	0 (0.0%)	0.008***
**Gestational age at delivery, weeks + days**						0.009**
Mean (w + d) ± SD (d)	37 + 2 ± 24	34 + 4 ± 26	37 + 5 ± 5	37 + 4 ± 25	38 + 6 ± 17	
Minimum - maximum	26 + 4 - 42 + 0	27 + 4 - 40 + 3	36 + 5 - 39 + 0	26 + 2 – 41 + 0	34 + 1 - 42 + 0	

^#^ normotensive IUGR only.

* = ANOVA.

** = Kruskal–Wallis test.

*** = Chi-Square test.

n.a. = not available, PE = Preeclampsia, GH = Gestational hypertension, IUGR = Intrauterine growth restriction, SGA = Small for gestational age, LGA = Large for gestational age.

### Prediction of PE by UtADV, PlGF, sFLT-1, and the sFLT-1/PlGF ratio

**Figure 1:**
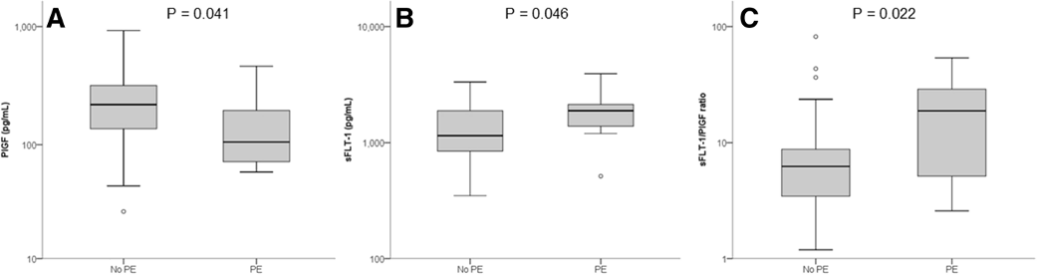
**Prediction of PE.** Boxplot analysis of maternal serum PlGF **(A)** and sFLT-1 **(B)** levels and their ratio **(C)**. Patients with development of preeclampsia (PE, n = 12) were compared with all patients without PE (n = 38). Boxes show the median (black line), and 25^th^, and 75^th^ percentiles (top and bottom lines of the box). The lines outside the box represent minimum and maximum values. Circles are outliers (>1.5× from the interquartile range). P values were computed by Mann–Whitney statistical tests.

The prevalence of PE in our high-risk study collective was 24.0% (12/50), with 75.0% (9/12) early-onset and 25.0% (3/12) late-onset diseases. PE was also present with chronic hypertension in four cases (all early onset). One patient developed concomitant HELLP syndrome. For prediction of PE, bilateral UtADV showed a PPV of only 24.0% with a false-positive rate (FPR) of 76.0%. Performance of ROC analysis using the mean PI of the uterine arteries showed an AUC of 0.630. At the optimal cut-off with a mean uterine PI ≥1.86, the specificity increased to 72.2%, with a PPV of 38.2%. However, the false negative rate also increased to 45.5%, resulting in a predictive accuracy of only 70.0%. In contrast, serum levels of PlGF and sFLT-1, as well as the sFLT-1/PlGF ratio, clearly improved discrimination between patients with and without PE (Figure 1, Table 2). If values were greater than the 95^th^ centile of gestation, the sFLT-1/PlGF ratio showed most useful test characteristics for PE, with a sensitivity of 66.7% and an FPR of only 10.5% (Table 3). The corresponding PPV was 66.7%, with an NPV of 89.5% and a predictive accuracy of the ratio of 84.0%. An elevated sFLT-1/PlGF ratio greater than the 95^th^ centile was associated with an increased risk for development of PE (OR 17.0; 95% confidence interval [CI] 3.5–83.0, P < 0.001) (Table 3). Results remained significant after weighting of parameters in multiple regression analysis with an adjusted OR for PE (adjusted OR 14.1; 95% CI 1.1–182.6, P = 0.043).

**Table 2:** **Maternal serum PlGF and sFLT-1 levels and their ratio**

	PE (n = 12)	No PE (n = 38)	P-value ^a^	Others (n = 16) (Uncomplicated)	GH (n = 16)	P-value ^b^	Normotensive IUGR (n = 6)	P-value ^c^	Delivery < 34 weeks (n = 7)	Delivery ≥ 34 weeks (n = 44)	P-value ^d^
PlGF (pg/mL)	105.6 (70.7-248.7)	218.5 (131.2-322.5)	0.041	274.6 (157.9-413.6)	198.7 (91.5-305.3)	0.142	165.9 (72.1-220.7)	0.033	75.1 (64.6-131.7)	221.7 (117.4-336.4)	0.003
sFLT-1 (pg/mL)	1879.0 (1379.5-2168.3)	1144.0 (842.2-1882.8)	0.046	1458.0 (1018.8-1896.3)	1032.6 (826.8-1649.8)	0.258	1324.4 (673.1-2012.0)	0.768	2158.0 (1858.0-3463.0)	1298.0 (846.1-1876.0)	0.008
sFLT-1/PlGF ratio	18.8 (4.8-29.1)	6.2 (3.4-8.8)	0.022	5.9 (2.9-8.6)	5.9 (3.6-9.1)	0.366	8.6 (4.0-19.6)	0.161	29.4 (14.1-53.9)	6.0 (3.4-9.0)	0.001

Data are given as median (interquartile range).

^a^ Mann–Whitney statistical test comparing PE vs No PE.

^b^ Mann–Whitney statistical test comparing GH vs Others.

^c^ Mann–Whitney statistical test comparing IUGR vs Others.

^d^ Mann–Whitney statistical test comparing delivery < 34 weeks vs delivery ≥ 34 weeks.

PE = Preeclampsia, GH = Gestational hypertension, IUGR = Intrauterine growth restriction.

**Table 3:** **Diagnostic indices of maternal PlGF, sFLT-1, and their ratio for identifying patients who will develop preeclampsia, hypertension without proteinuria, normotensive IUGR, and induced delivery at <34 weeks of gestation**

		Chi-Square P-value	Sensitivity	Specificity	PPV	NPV	LR + (95% CI)	LR- (95% CI)	OR (95% CI)
**PE (n = 12)**	PlGF	0.027	58.3%	78.9%	46.7%	85.7%	2.77 (1.27-6.04)	0.53 (0.27-1.05)	5.25 (1.31-21.03)
	sFLT-1	0.240	16.7%	94.7%	50.0%	78.3%	3.17 (0.50-20.13)	0.88 (0.68-1.15)	3.60 (0.45-28.86)
	sFLT-1/PlGF ratio	<0.001	66.7%	89.5%	66.7%	89.5%	6.33 (2.31-17.38)	0.37 (0.17-0.84)	17.0 (3.50-83.02)
**Gestational hypertension (n = 16)**	PlGF	0.514	37.5%	73.5%	40.0%	71.4%	1.42 (0.61-3.30)	0.85 (0.55-1.31)	1.67 (0.47-5.92)
	sFLT-1	1.000	6.3%	91.2%	25.0%	67.4%	0.71 (0.08-6.29)	1.03 (0.87-1.21)	0.69 (0.067-7.19)
	sFLT-1/PlGF ratio	0.292	12.5%	70.6%	16.7%	63.2%	0.43 (0.11-1.72)	1.24 (0.93-1.65)	0.34 (0.07-1.79)
**IUGR (n = 6)**	PlGF	0.654	16.7%	68.2%	6.7%	85.7%	0.52 (0.08-3.30)	1.22 (0.81-1.84)	0.43 (0.05-4.02)
	sFLT-1	1.000	0.0%	90.9%	0.0%	87.0%	0.00	1.10 (1.00-1.21)	0.00
	sFLT-1/PlGF ratio	1.000	16.7%	75.0%	8.3%	86.8%	0.67 (0.10-4.29)	1.11 (0.75-1.65)	0.60 (0.06-5.71)
**Induced delivery < 34 weeks (n = 7)**	PlGF	0.176	57.1%	74.4%	26.7%	91.4%	2.23 (0.98-5.07)	0.58 (0.34-1.38)	3.88 (0.75-20.12)
	sFLT-1	0.089	28.6%	95.4%	50.3%	89.1%	6.14 (1.03-36.79)	0.75 (0.47-1.20)	8.20 (0.94-71.73)
	sFLT-1/PlGF ratio	<0.001	85.7%	86.1%	50.1%	97.4%	6.14 (2.76-13.69)	0.17 (0.03-1.02)	37.00 (3.76-363.91)
**Early-onset PE (n = 9)**	PlGF	0.220	50.0%	73.8%	26.7%	88.6%	1.91 (0.81-4.51)	0.68 (0.33-1.39)	2.82 (0.60-13.24)
	sFLT-1	0.115	25.0%	95.2%	50.0%	87.0%	5.25 (0.86-32.02)	0.79 (0.53-1.18)	6.67 (0.79-56.64)
	sFLT-1/PlGF ratio	0.001	75.0%	85.7%	50.0%	94.7%	5.25 (2.26-12.18)	0.29 (0.09-0.98)	18.00 (2.92-110.96)
**Late-onset PE (n = 3)**	PlGF	0.075	75.0%	73.9%	20.0%	97.1%	2.88 (1.36-6.06)	0.34 (0.06-1.86)	8.50 (0.81-89.75)
	sFLT-1	1.000	0.0%	91.3%	0.0%	91.3%	0.00	1.10 (1.00-1.20)	0.00
	sFLT-1/PlGF ratio	0.240	50.0%	78.3%	16.7%	94.7%	2.30 (0.75-7.07)	0.64 (0.24-1.72)	3.60 (0.46-28.86)

Cut-off levels were defined as < 5^th^ centile for PlGF and > 95^th^ centile for sFLT-1 and the sFLT-1/PlGF ratio according to gestational specific reference values.

PE = Preeclampsia, GH = Gestational hypertension, IUGR = Intrauterine growth restriction, PPV = Positive predictive value, NPV = Negative predictive value, OR = Odds ratio, LR+/- = positive/negative Likelihood ratio.

**Figure 2:**
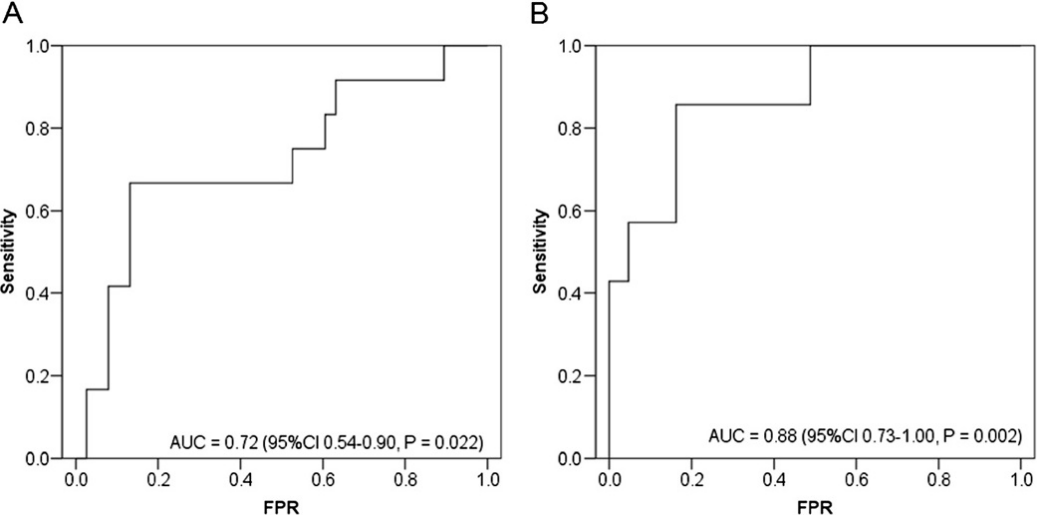
**ROC curves for prediction of PE (A) and induced delivery at <34 weeks of gestation (B) by using the sFLT-1/PlGF ratio.**

The sensitivity increased to 75.0% if only early-onset PE was included, but FPR also increased to 14.3%. We found four false-positive cases, where one patient showed a normotensive IUGR and two had hypertension without proteinuria (one with concomitant IUGR and one with intrauterine foetal death at 26 weeks of gestation). Two out of the four false-negative patients developed early-onset PE (one patient with concomitant HELLP syndrome at <34 weeks of gestation) and two developed late-onset PE (with only a mild clinical course in one patient). The fourth patient with false-negative serum markers developed late-onset PE, but fulfilled the criteria of severity with hypertension >160/110 mmHg. ROC analysis showed an AUC of 0.721 for the sFLT-1/PlGF ratio, with the best predictive cut-off of 13.0, resulting in a sensitivity of 66.7% with an FPR of 13.2% (Figure 2, Table 4). If a fixed FPR of 10% was assumed, the cut-off level of the ratio changed to 22.4, with a sensitivity of only 41.7%.

**Table 4:** **Results of receiver operating characteristic curve analysis with AUC values and cut-off levels of maternal serum PlGF (pg/mL) and sFLT-1 (pg/mL) levels and their ratio, resulting in optimal sensitivity and specificity**

		AUC	Cut-off	Sensitivity	Specificity	PPV	NPV
PE (n = 12)	PlGF	0.697	133.8	75.0%	76.3%	50.0%	90.6%
	sFLT-1	0.693	1171.5	91.7%	52.6%	37.9%	95.3%
	sFLT-1/PlGF ratio	0.721	13.0	66.7%	86.8%	61.5%	89.2%
EO PE (n = 9)	sFLT-1/PlGF ratio	0.735	13.0	75.0%	83.3%	51.4%	93.4%
Gestational hypertension (n = 16)	PlGF	0.494	117.6	37.5%	70.6%	37.5%	70.6%
	sFLT-1	0.364	590.5	100.0%	11.8%	34.8%	100.0%
	sFLT-1/PlGF ratio	0.456	2.3	100.0%	8.8%	34.0%	100.0%
IUGR (n = 6)	PlGF	0.640	262.3	100.0%	38.6%	18.2%	100.0%
	sFLT-1	0.439	1751.0	50.0%	63.6%	15.8%	90.3%
	sFLT-1/PlGF ratio	0.564	8.0	66.7%	56.8%	17.4%	92.6%
Induced delivery < 34 weeks (n = 7)	PlGF	0.834	133.8	85.7%	72.1%	33.3%	96.9%
	sFLT-1	0.807	1850.5	85.7%	74.4%	35.3%	97.0%
	sFLT-1/PlGF ratio	0.877	13.0	85.7%	83.7%	46.1%	97.3%

PE = Preeclampsia, EO = Early-onset, IUGR = Intrauterine growth restriction, ID = Induced delivery, PPV = positive predictive value, NPV = negative predictive value.

### Prediction of hypertension without proteinuria, IUGR, and induced delivery at <34 weeks of gestation

**Figure 3:**
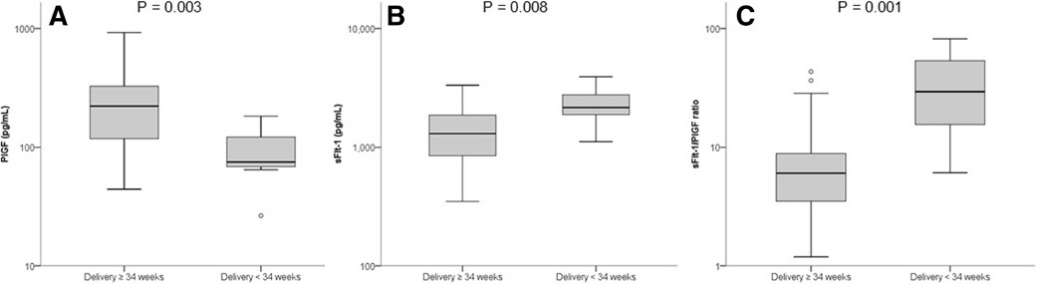
**Prediction of preterm delivery < 34 weeks. Boxplot analysis of maternal serum PlGF (A) and sFLT-1 (B) levels and their ratio (C).** Patients with delivery at <34 weeks of gestation (n = 6) were compared with the remaining patients (n = 44). Boxes show the median (black line), and 25^th^ and 75^th^ percentiles (top and bottom lines of the box). The lines outside the box represent minimum and maximum values. Circles are outliers (>1.5× from the interquartile range). P values were computed by Mann–Whitney statistical tests.

Results were further analysed in respect to development of hypertension without proteinuria and IUGR. As shown in Table 2, serum levels of PlGF were significantly lower in cases of IUGR compared to uncomplicated controls (P = 0.033), but failed significance if used as predictive parameter throughout the whole study population (Table 3). However, we found significant differences of the angiogenic markers if results were analysed in relation to gestational age at delivery (Figure 3). Measurements of sFLT-1 and PlGF were useful predictive markers for delivery at <34 + 0 weeks of gestation. If ROC analysis was performed, the AUC for the sFLT-1/PlGF ratio was 0.877, with a best cut-off of 13.0 (Figure 2). This cut-off resulted in a sensitivity of 85.7% with an FPR of 16.3%. If gestational age-dependent threshold levels greater than the 95^th^ centile were used, the FPR improved to 13.9% with the same sensitivity. The PPV and NPV were 50.1% and 97.4%, respectively. The ratio of sFLT-1/PlGF showed a predictive accuracy of 86.0%. An elevated sFLT-1/PlGF ratio greater than the 95^th^ centile was associated with an increased risk for delivery at <34 + 0 weeks (OR 37.0; 95% CI 3.8–363.9, P < 0.001) (Table 3). Results remained significant after weighting of parameters in multiple regression analysis with an adjusted OR (34.6; 95% CI 1.2–1026.2, P = 0.041).

## Discussion

### Main findings

Patients with an abnormal UtADV showed distinct foetal and maternal morbidity, with a high prevalence of PE, IUGR, and hypertensive disorders without proteinuria. However, only one of four patients with abnormal UtADV developed PE. Additional measurement of the sFLT-1/PlGF ratio was able to decrease the FPR from 76.0% to 10.5%. A negative test result below the 95^th^ gestational age-specific centile showed an NPV of 89.5%. The NPV was further improved if only patients with early onset PE (94.7%) or those who required delivery at <34 weeks (97.4%) were considered. Therefore, women with a normal sFLT-1/PlGF ratio were at low risk for development of one of these severe clinical courses. Additional measurement of sFLT-1 and PlGF would be helpful to reassure the majority of patients with an abnormal UtADV. An increased angiogenic ratio was associated with an increased risk of a severe hypertensive disorder and concomitant preterm delivery. Provident corticosteroid treatment of these women should be considered.

### Interpretation

There are different approaches for prediction of PE. Doppler ultrasound of the uterine arteries during the second trimester is the best-known single predictor for PE, with a detection rate up to 80% for early-onset disease [14, 24–26]. However, the majority of patients with abnormal UtADV will never develop PE, which suggests the need to improve the predictive accuracy. Therefore, use of a well-established and widely performed test for identification of a high risk collective and its combination with measurement of angiogenic factors for improvement of specificity is a practically oriented approach. The use of a fully automated diagnostic system that analyses sFLT-1 and PlGF guarantees a high test quality. The Elecsys® immunoassays show high precision and reliability, and allow execution of measurements in laboratories for routine clinical diagnosis [21].

Recent studies have reported complex algorithms that allow prediction of PE by integration of miscellaneous risk factors, including analysis of angiogenic factors between 11–13 weeks of gestation [27–29]. Identification of high-risk patients during the first trimester offers the advantage of a preventive therapy with low-dose aspirin, which more than halves the risk of severe PE [30]. However, the ability of prediction or exclusion of PE during the second trimester may also be useful because it can facilitate decision making as to whether intensified patient’s care or corticosteroid treatment for foetal lung maturation will be necessary. There is also increasing evidence for effective preventive treatment strategies in high-risk patients during the second trimester [31–33]. Various (nested) case–control studies have examined the differences in maternal angiogenic protein concentrations during the second trimester preceding the occurrence of PE [8, 34–41]. The usefulness of circulating sFLT-1 and PlGF as a test to predict PE in healthy pregnant women was recently proven in a comprehensive prospective cohort study by McElrath and colleagues [11]. In a cohort of 2243 patients, the plasma levels of angiogenic factors were repeatedly measured at 10, 17, 24, and 35 weeks of gestation. In the study sample, 139 (6.2%) patients developed PE. At 10 weeks of gestation, the authors did not find any significant differences between plasma concentrations of PlGF or sFLT-1, or the sFLT-1/PIGF ratio in patients with PE compared with healthy controls. Differences became significant at 17 weeks for PlGF (P = 0.009) alone, and were highly significant for PlGF and the sFLT-1/PIGF ratio at 24 weeks (P < 0.001). In contrast, differences for sFLT-1 became significant at 35 weeks. At 24 weeks, the highest detection rate (62.2%) was observed for PlGF alone, with an FPR of 38.4% and a PPV of 9.5%. The relative risk for development of PE was 2.46 (95% CI 1.66–-3.65) if a cut-off level of 379 pg/mL was used [11]. Similar results were shown in a further prospective study including 1622 healthy Hispanic women [25]. Maternal plasma concentrations of various angiogenic proteins were measured at 6–15 and 20–25 weeks of gestation. During pregnancy, 3.8% of patients developed PE (n = 62), and 0.6% had early-onset PE (n = 9). In midtrimester, PlGF measurements alone showed a better test performance (P < 0.001) than sFLT-1 (P = 0.5) or the sFLT-1/PIGF ratio (P = 0.006). However, the detection rate for PlGF was only 51.6%, with an FPR of 23.6% and a PPV of 8%. A reasonable cut-off value was 215 pg/mL, resulting in an OR of 3.8 (95% CI 2.2–6.7). The detection rate for early-onset PE was 100%, and was therefore better, with an FPR of only 4.2% at a cut-off level of 126 pg/mL [25]. The authors of both studies concluded that angiogenic protein concentrations alone, measured in early or even late pregnancy, were of limited utility for prediction of PE. For prediction of PE, its low incidence and concomitant low predictive value are problematic. Therefore, identification of high-risk patients may be an alternative, more suitable approach. In a prospective cohort study of 3348 patients, Espinoza and colleagues examined the relationship between UtADV and plasma concentrations of PlGF and sFLT-1 between 22 and 26 weeks of gestation [24]. In this study, they showed that abnormal UtADV and maternal PlGF were independent explanatory variables for the occurrence of PE. For prediction of PE, the FPR of abnormal UtADV alone was low (10.4%). Abnormal UtADV had a better test performance than maternal PlGF for identification of a high-risk collective, with ORs for all types of PE and early-onset PE of 4.3 (95% CI 2.82–6.66) and 24.1 (95% CI 9.61–60.44) compared with PlGF (cut-off, 280 pg/mL), with corresponding ORs of 2.6 (95% CI 1.57–3.94) and 5.5 (95% CI 1.98–15.08). The combination of both parameters increased the OR for early-onset PE to 43.8 (95% CI 18.48–103.9). The PPV for early-onset PE was improved from 5.4% for abnormal UtADV and 1.4% for PlGF alone to 12.7% if both factors were combined with a positive likelihood ratio of 18.48 (95% CI 13.07–26.13). This study [24] impressively showed that PlGF significantly contributed to the prediction of patients who were going to develop early-onset PE, and thus confirmed the results of a smaller study by Stepan and colleagues [42]. The supplemental table compares the test characteristics of the sFLT-1/PlGF ratio of various studies (Additional file 1: Table S1). The prospective cohort study by Moore Simas and colleagues included only patients with at least one risk factor for PE in their medical history [43]. In this study, the determination of sFLT-1 showed a good predictive accuracy for early-onset PE, with an AUC value of 0.90 (95% CI 0.78–1.0) between 22 and 26 weeks of gestation. The sFLT-1/PlGF ratio was even better with an AUC value of 0.97 (95% CI 0.91–1.0). Similar results of other studies, including our own data, showed an increase in specificity if the sFLT-1/PlGF ratio instead of PlGF alone was used for prediction of early-onset PE. However, compared with a decrease in PlGF, an increase in sFLT-1 in patients with PE is a late event, with only a narrow time frame between diagnosis and clinical manifestation of PE [8, 11].

Although the end-point of our study was the development of PE, we additionally analysed the correlation between angiogenic factors and the occurrence of other adverse outcome parameters, such as IUGR and delivery at <34 weeks. The group of induced deliveries showed great overlap with the PE cases and could be regarded as a surrogate for a severe clinical course. Therefore, identification of these patients is of high clinical relevance. With an AUC of 0.88, we observed a satisfactory test performance for prediction of cases with a delivery at <34 weeks of gestation. Although PPV was only 50.1%, we found an NPV of 97.4%. In patients with abnormal UtADV and a normal angiogenic ratio, the need for delivery at <34 weeks following hypertensive pregnancy disorders is highly unlikely. With a sensitivity of 71% and a specificity of 76%, Stepan and colleagues also reported the capability of the sFLT-1/PlGF ratio for prediction of preterm delivery [42]. Additionally, in a prospective study of patients with suspected early-onset PE, the sFLT-1/PlGF ratio showed prognostic relevance [44]. Patients with a ratio ≥85 had a significantly increased risk for delivery within the next 2 weeks, with a hazard ratio of 15.2 (95% CI 8.0–28.7).

In our study, angiogenic factors were not suitable for prediction of hypertension without proteinuria or for normotensive IUGR, although in IUGR, the levels of PlGF were significantly lower compared with inconspicuous controls. Differences reported in other studies were also only moderate, and not helpful for diagnosis or prediction of these adverse pregnancy outcome parameters [12, 41, 42].

## Conclusion

In high-risk patients, measurement of angiogenic factors shows a good predictive power for development of PE. The best results can be achieved using the ratio of sFLT-1 and PlGF, with a predictive test accuracy of 84%. In patients with abnormal UtADV, a high FPR of Doppler measurement alone is improved to 10.5%. An increased sFLT-1/PlGF ratio is also predictive for patients who need delivery at <34 + 0 weeks of gestation. The test performed even better for prediction of preterm delivery compared with PE, with a sensitivity and specificity of >80%. In particular, a NPV of 97.4% can help to reassure most patients with an abnormal UtADV.

## Electronic supplementary material

Additional file 1: Table S1: Test indices of the midtrimester sFlt-1/PlGF ratio for prediction of preeclampsia. Comparison of relevant studies (see Discussion). (DOC 37 KB)
