# Supplementary material for: Prediction of preeclampsia and induced delivery at <34 weeks gestation by sFLT-1 and PlGF in patients with abnormal midtrimester uterine Doppler velocimetry: a prospective cohort analysis
Source: BMC Pregnancy Childbirth. 2014 Aug 28;14:292. doi: 10.1186/1471-2393-14-292 (PMC4162945; doi:10.1186/1471-2393-14-292)
Supplement: Supplementary file 1 — Additional file 1: Table S1: Test indices of the midtrimester sFlt-1/PlGF ratio for prediction of preeclampsia. Comparison of relevant studies (see Discussion). (DOC 37 KB) [file 12884_2014_1174_MOESM1_ESM.doc]

**Additional file 1: Table S1.** Test indices of the midtrimester sFlt-1/PlGF ratio for prediction of preeclampsia. Comparison of relevant studies (see discussion).

| **Study, Author,Year** | **Time of**  **measurement (weeks)** | **Patients (n)** | **Mean/**  **median ratio** | **Control (n)** | **Mean /median ratio** | **P-value** | **DR (%)** | **FPR (%)** | **Cut-off** | **OR (95%CI)** | **LR+ (95%CI)** | **LR- (95%CI)** | **Comment** |
| --- | --- | --- | --- | --- | --- | --- | --- | --- | --- | --- | --- | --- | --- |
| Stepan H et al. Hypertension 2007 | 19-24 | 12 (all PE) 9 (PE with delivery <34 wks) | n.a. | 38 | 3.6 | <0.05 | 62 67 | 49 49 | 3.15 3.15 |  |  |  | prospective cohort study with abnormal UtADV |
| Kusanovic JP et al. J Maternal Fetal Neonat Med 2009 | 20-25 | 62 (all PE) 9 (EO PE) | 5.88 100 | 1560 1613 | 4.76 5 | 0.006 <0.001 | 40.3 100 | 21.5 10.9 | 8.3 12.2 | 2.0 (1.3-3.4) | 1.9 (1.2-3.5) 9.2 (6.2-9.2) | 0.8 (0.6-0.9) 0.0 (0.0-0.3) | prospective cohort study, low-risk patients |
| Moore Simas TA et al. AJOG 2007 | 22-26 | 5 (EO PE) 7 (LO PE) | 5.6 1.6 | 82 | 1.1 | <0.05 |  |  |  |  |  |  | high-risk group, prospective cohort |
| Villa PM et al. BMC Pregnancy Childbirth 2013 | 18-20 | 6 (EO PE) 21 (LO PE) | 15.9 6.7 | 26 | 6.9 | n.s. n.s. |  |  |  |  |  |  | nested case control, high-risk controls |
| McElrath TF et al. AJOG 2012 | 24 | 139 (PE) | 18 | 1951 | 12.9 | <0.0001 | 52.8 | 38.6 | 16.6 | 1.67 (1.14-2.44) |  |  | prospective cohort study, low-risk patients |
| Hassan MF et al. Obstet Gynecol Int 2013 | 15-20 | 80 (PE) | 44.6 | 80 | 14.4 | <0.0001 | 91.6 | 13.6 | 24.5 | 67 (29.3-162.1) | 6.7 (4.9-9.3) | 0.1 (0.05-0.2) | nested case control study |
| Stubert J et al. (presented data) | 19-26 | 12 (all PE) 9 (EO PE) | 21.5 | 38 | 10.4 | 0.022 | 66.7  75.0 | 10.5  14.3 | 13.0*  13.0* | 17.0(3.5-83.0)  18.0 (2.9-111.0) | 6.3 (2.3-17.3)  5.3 (2.3-12.2) | 0.4 (0.2-0.8)  0.3 (0.1-1.0) | prospective cohort study with abnormal UtADV |

* calculated cut-off values only, test indices based on gestational age dependent values for the ratio > 95^th^ centile

PE = Preeclampsia, EO = Early-onset, LO = Late-onset, DR = Detection rate, FPR = False-positive rate, OR = Odds ratio, LR+/- = positive/negative Likelihood ratio
